# Special Analytical and Health Commission on Tinned and Preserved Food

**Published:** 1906-08-25

**Authors:** 


### SPECIAL ANALYTICAL AND HEALTH COMMISSION ON TINNED AND PRESERVED FOOD.

### VIII. SAUCES AND PICKLES.

### Appetite and Hunger.

Chemists may analyse food, and physiologists may experiment with regard to its nutritive value, but it is the human subject himself who must eat, swallow, and digest. From this it follows that in the digestion of food there is always a personal factor which refuses to be weighed upon a chemical balance or recorded by any graphic method. An essential feature of this personal element is appetite, and its first cousin is hunger. Those who read their Shakespeare with an eye to the truths of modern physiology have probably been struck not only by the literary elegance, but also by the scientific depth of the courteous preprandial wish, "May good digestion wait on appetite, and health on both." At the end of the last century the Russian physiologist Pawlow told us that the two most important factors in causing the secretion of an adequate supply of gastric juice for the digestion of, say, a mixed meal, were appetite and chemical stimuli derived from the food and capable of acting upon the sensory end-organs of the gastro-intestinal tract. In a series of elaborate researches he shows with beautiful clearness how different the physiological mechanism of the secretion of the gastric and pancreatic juice is to that of the saliva. Almost any stimulus, whether chemical or mechanical, will, when applied to the mucous membrane of the mouth, cause secretion of saliva, whereas specific stimuli are required to call forth the secretion of types of gastric and pancreatic juice endowed with special digestive qualities, *vis-à-vis* definite nutriments. Further, not only can the secretions of these juices be propitiated by certain psychical and chemical stimuli, but it can be also entirely inhibited by stimuli of the same class. The digestion of even the strongest and least æsthetic of us cannot be wholly independent of the appearance and taste of food. Appetite, if it cannot be absolutely created by art, certainly can be nurtured, and also entirely destroyed, by art. Appetite may perhaps best be defined in the words of Brillat-Savarin as the first impression the individual receives that he wants to eat. This feeling is, by the association of ideas, invariably connected with memories of taste sensations. Directly one begins to want to eat one begins to remember and think of tasty things one has eaten. If the hint to eat given by appetite is not taken nature reinforces the sensation and appetite gives place to hunger. Hunger must be regarded as pathological, and we are by no means sure that actual hunger propitiates a secretion of the digestive juices; in fact, what we know at present upon the subject rather indicates that its action in this direction is an inhibitory one. The field of origin of the different afferent impulses which our sensorium presents to us as appetite is probabiy the gastro-intestinal or splanchnic area. It is, on the other hand, probably rightly suggested that the sensation of hunger is derived from the tissues generally and is essentially their craving for food. How appetite may pass into hunger, and the symptoms which manifest themselves during this transition, as well as the inhibitory influence of hunger upon digestion, are well described by Brillat-Savarin in his amusing and instructive book, "La Physiologie du Gout."

### Taste, Condiments, and Spices.

The importance of taste in creating and fostering appetite and hence in promoting digestion cannot be over-estimated. Stimuli proceeding entirely from the psychical side may produce, probably by means of the association of ideas, sensations of taste, but the common stimuli of this class with which we have to deal are chemical ones. Concerning their actual chemical nature we often know little. Most foods contain in their natural condition substances having this effect; some, however, do not. Egg-white, for instance, according to Pawlow, is not capable of exciting the secretion of gastric juice; simple fat seems to actually inhibit it. This latter is probably the reason why butter is the most wholesome of all fats because it contains high fatty acids which give it a bouquet and thus prevent it from inhibiting the secretion of the digestive juices. The average man does not, however, trust to the secretion-stimulating substances originally present in his food, but reinforces them by a class of agent which we term condiment. By a condiment we mean a substance which, although not strictly speaking a food, in that it does not supply energy to the body, yet nevertheless promotes the digestion of food by stimulating through the organs of taste and also probably in other ways the secretion of the digestive juices.

The receipts of the well-known sauces have been the result of much experience and experiment, and are just as much to be respected as the receipts of the well-known liqueurs, such, for instance, as Chartreuse.

### Samples Received.

We have received from Messrs. Lazenby and Son a sample of their essence of anchovies. The taste of anchovies is well known, and this preparation has a remarkably good taste, and it gives evidence of being carefully prepared from well-selected anchovies. A very small quantity of the essence will give the flavour to a large amount of neutral sauce. Another point in its favour is that it is not too salt, which is very apt to be the case in preparations of this kind. Messrs. Sutton, Sons and Company, of Osborne Works, King's Cross, London, N., have also submitted a sample of their essence of anchovies. This essence is prepared from Georgona fish by a new process, and has been examined chemically by competent authorities, and found to be well up to standard. For our part we have no hesitation in recommending Messrs. Suttons' product as being a pure and in every way desirable essence of anchovies. Messrs. Lazenby have sent us a bottle of their Picalilli and also one of their mixed pickles.

The vegetables in these pickles are crisp and of good quality and flavour, and the sauce of the Picalilli is stimulant and appetising. We think there is little doubt that, from the gastronomic standpoint, pickles of this nature, when taken in proper quantities, are distinct adjuvants to cold meats. We have to thank Messrs. Gordon and Dilworth for a bottle of their mushroom catsup. The mushroom, with its near relation the truffle, possesses a flavour much prized in the culinary art. The catsup before us has a distinctly good flavour of mushrooms, and this flavour is not masked, as is often the case in these preparations, with salt and spices. The same firm have sent us a bottle of their tomato catsup. This sauce is prepared from fresh whole tomatoes, is of good colour and flavour, and keeps well when opened. We have received from Messrs Hoe and Company a bottle of the celebrated Hoe's sauce. This preparation has a piquant and appetising taste, and, if used in the right strength, adds a distinct relish to meat, game, or fish. Messrs. Lea and Perrin, the well-known makers of Worcestershire sauce, have sent us a bottle of this world-renowned relish. Tastes differ, but we venture to think that the widespread use of this sauce is entirely justified, and that, although perhaps the consumption of it in the West is less than that of Shoya in the East, of which it is computed that each member of the population consumes one fluid ounce a day, yet nevertheless it may truly be said of it that it has increased the appetite of millions. Messrs. Sutton, Sons and Company have submitted to us a bottle of their Victory dinner relish and of their Worcestershire sauce. We consider that both these products are to be recommended. Messrs. Goodall, Backhouse and Company, of Leeds, have kindly sent us a sample of their celebrated Yorkshire relish. We all know and have used this product. The examination to which we have submitted it in the course of our present inquiry justifies us in concluding that it is thoroughly wholesome and carefully made from choice ingredients. Messrs. Lazenby have forwarded to us a sample of their original Harvey's sauce. This sauce is made from the original receipt, and possesses an exceedingly fine and appetising flavour. We have received a sample of digestive sausage relish from Mr. William Harris (Smithfield brand). This sauce is specially prepared as a relish for sausages, and the trial that we have made with it confirms the view that it is well suited, both by its flavour and digestive properties, to be eaten with sausages.

